# Symmetry-Induced Structuring of Ultrathin FeO and Fe_3_O_4_ Films on Pt(111) and Ru(0001)

**DOI:** 10.3390/nano8090719

**Published:** 2018-09-12

**Authors:** Natalia Michalak, Zygmunt Miłosz, Gina Peschel, Mauricio Prieto, Feng Xiong, Paweł Wojciechowski, Thomas Schmidt, Mikołaj Lewandowski

**Affiliations:** 1 Institute of Molecular Physics, Polish Academy of Sciences, M. Smoluchowskiego 17, 60-179 Poznań, Poland; michalak@ifmpan.poznan.pl (N.M.); pwojciechowski@ifmpan.poznan.pl (P.W.); 2 NanoBioMedical Centre, Adam Mickiewicz University, Umultowska 85, 61-614 Poznań, Poland; zmilosz@amu.edu.pl; 3 Fritz-Haber-Institut der Max-Planck-Gesellschaft, Faradayweg 4-6, 14195 Berlin, Germany; peschel@fhi-berlin.mpg.de (G.P.); prieto@fhi-berlin.mpg.de (M.P.); xxf2012@mail.ustc.edu.cn (F.X.); schmidtt@fhi-berlin.mpg.de (T.S.); 4 Department of Chemical Physics, University of Science and Technology of China, No. 96, JinZhai Road Baohe District, Hefei 230026, China

**Keywords:** iron oxides, FeO, Fe_3_O_4_, ultrathin films, epitaxial growth, platinum, ruthenium, symmetry, LEEM, LEED, XPEEM

## Abstract

Iron oxide films epitaxially grown on close-packed metal single crystal substrates exhibit nearly-perfect structural order, high catalytic activity (FeO) and room-temperature magnetism (Fe_3_O_4_). However, the morphology of the films, especially in the ultrathin regime, can be significantly influenced by the crystalline structure of the used support. This work reports an ultra-high vacuum (UHV) low energy electron/synchrotron light-based X-ray photoemission electron microscopy (LEEM/XPEEM) and electron diffraction (µLEED) study of the growth of FeO and Fe_3_O_4_ on two closed-packed metal single crystal surfaces: Pt(111) and Ru(0001). The results reveal the influence of the mutual orientation of adjacent substrate terraces on the morphology of iron oxide films epitaxially grown on top of them. On fcc Pt(111), which has the same mutual orientation of adjacent monoatomic terraces, FeO(111) grows with the same in-plane orientation on all substrate terraces. For Fe_3_O_4_(111), one or two orientations are observed depending on the growth conditions. On hcp Ru(0001), the adjacent terraces of which are ‘rotated’ by 180° with respect to each other, the in-plane orientation of initial FeO(111) and Fe_3_O_4_(111) crystallites is determined by the orientation of the substrate terrace on which they nucleated. The adaptation of three-fold symmetric iron oxides to three-fold symmetric substrate terraces leads to natural structuring of iron oxide films, i.e., the formation of patch-like magnetite layers on Pt(111) and stripe-like FeO and Fe_3_O_4_ structures on Ru(0001).

## 1. Introduction

Iron oxides exhibit unique physical and chemical properties that find applications in various industrial fields. The properties of iron oxide nanostructures, such as nanoparticles or thin films, differ from those of the corresponding bulk oxides, which is related to their limited dimensionality and – in the case of thin films—the interaction with the substrate on which they grow. It has been shown that ultrathin wüstite (FeO) films exhibit superior catalytic activity in the CO oxidation reaction [1,2], which is related, among other factors, to the strong film–substrate interaction [3]. Ultrathin (few-nanometers-thick) magnetite (Fe_3_O_4_) films, on the other hand, have been shown to exhibit ferro- (ferri-)magnetic ordering at room temperature [4,5,6], with magnetic properties dependent on the morphology of the films influenced by the structure of the used support [7].

**Figure 1:**
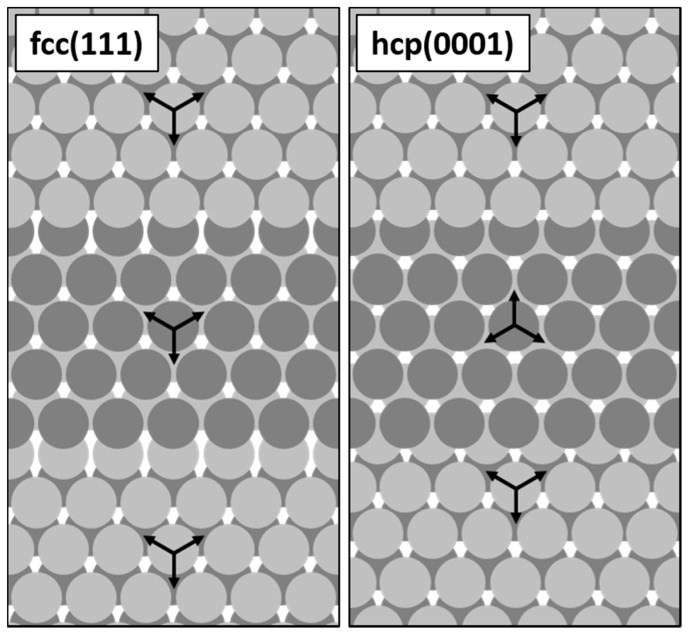
Schematic drawings showing the mutual orientation of adjacent monoatomic terraces on stepped fcc(111) and hcp(0001) surfaces (only the first two atomic layers are shown for each terrace; based on a similar figure published in Reference [10]).

Iron oxide films can be grown on various metal single crystal substrates, the two most commonly used of which are cubic Pt(111) [8] and hexagonal Ru(0001) [9]. Even though the surfaces of both of these substrates exhibit close-packed atomic planes with relatively similar lattice spacing, being 2.78 Å for Pt(111) and 2.71 Å for Ru(0001), their bulk crystal structures determine different in-plane orientation of adjacent atomic terraces on stepped surfaces. In the case of fcc platinum, the orientation is identical and results from the ABCABC-stacking. In the case of hcp ruthenium, the terraces are ‘rotated’ by 180° with respect to each other (when looking at the mutual positions of atoms in the first two atomic layers of each terrace [10]), which is a consequence of the ABAB-stacking. These differences are schematically shown in Figure 1.

On Pt(111), iron oxide films grow via the so-called Stranski–Krastanov (layer + islands) mode [8]. Firstly, an FeO layer is formed in direct contact with the metal support. Then, three-dimensional Fe_3_O_4_ islands start to nucleate on top of FeO. These islands may ultimately coalesce and form a closed magnetite film at a total thickness of >100 Å [8]. On Ru(0001), the growth mode depends on the preparation conditions: The use of O_2_-assisted Fe deposition onto a heated substrate results, similarly to the case of Pt(111), in the formation of Fe_3_O_4_(111) islands growing on top of FeO(111) [9]. Iron deposition onto a substrate kept at room temperature and post-oxidation, on the other hand, results in an additional thermodynamically-driven transformation of the FeO(111) film underneath Fe_3_O_4_(111) islands to magnetite (so that Fe_3_O_4_ grows directly on Ru(0001) [11]). FeO(111) itself preferably grows as an Fe-O monolayer (ML) on Pt(111) [8] (with the possibility of stabilizing up to 2.5 MLs under certain growth conditions) and as an Fe-O-Fe-O bilayer on Ru(0001) [9] (with the possibility of stabilizing a structurally ill-defined monolayer [11] or a 4 MLs-thick film [9] using certain preparation recipes). The oxide has a rock-salt structure and, when looking along ‹111› direction, consists of alternately stacked close-packed layers of Fe^2+^ and O^2-^ ions, with iron atoms located at the interstitial sites of the oxygen lattice [8]. Due to the lattice mismatch between the oxide and the support (FeO(111) has an in-plane lattice constant of around 3.1 Å), ultrathin FeO films are characterized by Moiré superstructures the fingerprints of which can be observed in scanning tunneling microscopy (STM) images and low energy electron diffraction (LEED) patterns [8,9]. The coincidence structures responsible for the formation of such superstructures on Pt(111) and Ru(0001) are 8 FeO units on 9 Pt units and 7 FeO units on 8 Ru units (resulting in 25 Å and 21.6 Å Moiré periodicities, respectively). Fe_3_O_4_(111), on the other hand, has an inverse spinel structure with a mixture of Fe^2+^ Fe^3+^ ions arranged in Kagomé-type and mix-trigonal layers separated by close-packed O^2-^ planes. The interatomic distances within the mix-trigonal lattices equal to the distances between unoccupied sites in the Kagomé lattices and are twice as large as those in the oxygen lattice (approx. 6 Å vs. 3 Å). This gives rise to the characteristic (2 × 2) LEED pattern and accounts for the atomic spacing seen in the STM images [12].

Low energy electron microscopy (LEEM) is a powerful tool that allows real-time observation of thin films growth with a nanometer-scale resolution. When equipped with an imaging energy analyzer and used with a synchrotron light as an excitation source, it can be operated in the X-ray photoemission electron microscopy (XPEEM) mode which allows obtaining chemical contrast on the acquired images. The instrument also gives the possibility to record micro-spot low energy electron diffraction (µLEED) patterns from the selected sub-micrometer-sized regions. A complete overview of the LEEM-based techniques can be found in [13].

LEEM was used by various research groups for the studies of iron oxide films on Pt(111), Ru(0001) and Ag(111) (see e.g. References [6,11,14,15,16,17,18]). The results provided information on the growth, structure, electronic and magnetic properties of the films at the nanometer-scale. However, none of these studies comprehensively addressed the influence of different substrates’ symmetry on the structure of iron oxide islands and films grown on top of them.

This work reports a comparative LEEM/XPEEM and µLEED surface science study of the growth and structure of FeO and Fe_3_O_4_ films on Pt(111) and Ru(0001). In addition to the well-known dependence of the film morphology on the growth conditions, the results reveal the influence of the substrates’ symmetry—more precisely: the mutual orientation of adjacent substrate monoatomic terraces—on the structure of iron oxide islands and films epitaxially grown on top of them. The established procedures allow the preparation of naturally structured wüstite and magnetite layers that may find potential applications in future spintronic devices.

## 2. Materials and Methods

The experiments were performed using the SMART instrument (built up within a collaboration of several German groups [19,20]) located at the BESSY II synchrotron of the Helmholtz-Zentrum Berlin (HZB) (beamline UE49-PGM-SMART). The instrument is an aberration-corrected and energy-filtered spectro-microscopy system operating under ultra-high vacuum (UHV; base pressure: 1 × 10^−10^ mbar). It combines real-time electron- and X-ray-based microscopy (LEEM, XPEEM) and electron diffraction (µLEED), being a perfect tool for the studies of the growth and structure of epitaxial thin films (see Refs. [19] and [20] for more details).

Pt(111) and Ru(0001) single crystals (purity 99.999%; from MaTeck GmbH, Jülich, Germany*)* were cleaned by repeated cycles of 1 keV Ar^+^ ions sputtering at room temperature, annealing in 1 × 10^−6^ mbar O_2_ at 800–1000 K (Ar and O_2_ 99.999% pure; from Westfalen AG, Münster, Germany) and in UHV at 1300 (Pt(111)) or 1450 K (Ru(0001)). The crystalline order and cleanliness of the substrates were monitored with LEEM, µLEED and XPS. Iron (99.995%; Alfa Aesar GmbH, Karlsruhe, Germany) was deposited using a commercial evaporator (Focus GmbH, Hünstetten, Germany) onto the substrates kept at room temperature and post-oxidized in 1 × 10^−6^ mbar O_2_ at 900 K for several minutes. The Fe deposition rate was determined from the obtained LEEM images (1 ML of iron is defined as the amount needed for the formation of a closed FeO(111) film on Pt(111); calibration accuracy: +/−5%). The temperature of the samples was controlled using an infrared pyrometer (LumaSense Technologies, Inc., Santa Clara, CA, USA).

The growth and structure of iron oxide films were characterized by LEEM, XPEEM and µLEED. The energies at which the data were taken are indicated in the captions of the figures. The LEEM-IV data presented in this work were obtained by plotting the intensity of a certain surface region from a series of images recorded at different beam energies, the dark field (DF) LEEM-IV curves from a series of dark field images acquired by mapping a particular diffraction spot, while the XPEEM-IV spectra from a stack of XPEEM images taken at different kinetic energies (the resulting XPEEM-IV curves, calibrated with respect to the valence band, are equivalent to micro-spot X-ray photoelectron spectroscopy (µXPS) spectra). Some sets of spectra were additionally normalized to allow for direct comparisons.

## 3. Results and Discussion

**Figure 2:**
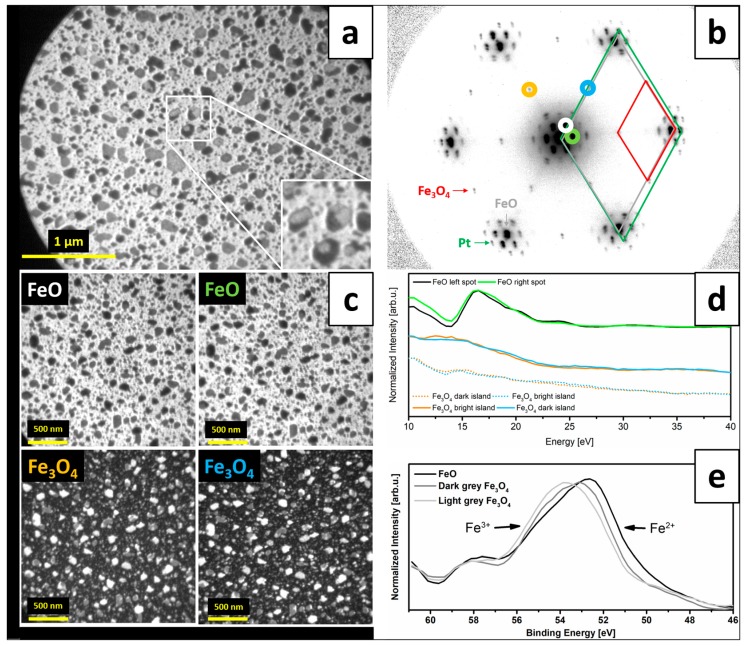
LEEM image (energy: 10 eV) (**a**) and µLEED pattern (energy: 42 eV) (**b**) of iron oxide structures grown on Pt(111) by ~1.9 MLs Fe deposition under UHV at RT and post-oxidation in 1 × 10^−6^ mbar O_2_ at 900 K; the Pt(111), FeO(111) and Fe_3_O_4_(111) unit cells are marked on the µLEED pattern with green, grey and red rhombuses, respectively; (**c**) presents DF-LEEM images obtained by using the diffraction spots of FeO(111) (imaging energy: 17 eV) and Fe_3_O_4_(111) (10 eV) (marked in colors in (**b**)), while (**d**) shows the DF-LEEM-IV curves plotted from stacks of different DF-LEEM images taken at different energies (the corresponding pairs of curves were normalized and the curves were offset for clarity); (**e**) presents the XPEEM-IV data (photon energy: 150 eV) plotted for various surface regions that correspond to different iron oxide structures.

Figure 2a presents a LEEM image of iron oxide structures grown on Pt(111) by ~1.9 MLs iron deposition under UHV and subsequent oxidation in 1 × 10^−6^ mbar O_2_ at 900 K. For these particular support, iron coverage and oxidation conditions, a formation of a closed FeO(111) film with nucleating Fe_3_O_4_(111) islands on top, was expected [8]. Interestingly, at certain energies, three different contrasts were observed on the acquired LEEM images, visible as light grey and dark grey hexagonal and irregularly-shaped islands on a bright background (these structures can be seen more clearly in the inset to Figure 2a).

The µLEED pattern taken from this surface is shown in Figure 2b. Contributions from several ordered surface structures could be identified: Six main (1 × 1) reflexes were assigned to the substrate Pt(111). Six additional spots, positioned closer to the (0;0) spot and representing a larger interatomic spacing, were assigned to originate from FeO(111). Satellite spots around the Pt(111) spots are known to result from the multiple scattering at the Moiré superstructure [8]. Additional (2 × 2) spots with respect to Pt(111)-(1 × 1) spots were tentatively assigned to originate from Fe_3_O_4_(111) [8,12]. The number and shape of the Moiré satellite spots indicated the presence of a bilayer (Fe-O-Fe-O) film [21]. The precise assignment of different surface species visible in LEEM was possible thanks to dark field images obtained using the diffraction spots originating from different surface structures (the images are presented in Figure 2c with a color-code that follows the marks on the µLEED pattern in Figure 2b). The results confirmed that the bright background is FeO(111), while the light grey and dark grey islands are Fe_3_O_4_(111). In general, FeO(111) and Fe_3_O_4_(111) have a three-fold rotational symmetry, which is the same as the substrate Pt(111), and, therefore, can potentially grow on a three-fold symmetric substrate in two domains rotated with respect to each other by 180°. It was indeed shown in Ref. [22] that FeO(111) can grow in such two domains on Pt(111), where one domain orientation is more favored than the other. However, closed films were found to exhibit only one (more favored) orientation, which is in line with the morphology observed in our experiments. Similarly, for Fe_3_O_4_(111), which grows on top of FeO(111)/Pt(111), two quantitatively inequivalent in-plane orientations were reported in Refs. [12] and [15] (as could be expected for three-fold symmetric Fe_3_O_4_(111) growing on three-fold symmetric FeO(111)). However, in our case, dark field imaging of the two neighboring Fe_3_O_4_(111) diffraction spots (i.e., the (2 × 2) spots with respect to Pt(111)-(1 × 1) spots) did not result in two different image contrasts, thus indicating that all Fe_3_O_4_ islands have the same in-plane orientation (Figure 2c). As the contrast on the DF-LEEM images may also depend on the beam energy, we recorded DF-LEEM-IV curves for two neighboring μLEED spots of each type, i.e., the FeO-Moiré spots and the Fe_3_O_4_-(2 × 2) spots (the resulting curves are displayed in Figure 2d and marked in colors corresponding to the rings in Figure 2b). In both cases, the corresponding curves had the same character, which confirmed the same orientation of FeO(111) and Fe_3_O_4_(111) on all substrate terraces. Notably, at certain energies, the FeO signal could also be observed at the positions of the light grey and dark grey islands. This indicated that FeO was present underneath Fe_3_O_4_ and that the magnetite islands were relatively thin (thinner than the probing depth of the DF-LEEM). The contrast in LEEM may, of course, also depend on complex interactions between different layers and has to be interpreted with proper caution, however, the observed growth seems reasonable, taking into account the deposited amount of iron and the existing knowledge on the structure of iron oxide films on Pt(111).

The recorded XPEEM data allowed us to plot XPEEM-IV curves from each type of region and determine the compositional differences between the FeO background and two types of Fe_3_O_4_ islands. The data recorded for the binding energy range where the Fe 3p signals are known to appear are shown in Figure 2e (we used the 3p signals instead of the commonly used 2p due to the specific set-up of the beamline in which lower photon energies result in higher intensity). The spectrum obtained by plotting the intensity from the whole field of view (not shown) revealed a broad and asymmetric peak, indicating superposition of components originating from iron in different oxidation states (i.e., Fe^2+^ and Fe^3+^, which occur at the low and high energy sides of the spectrum, respectively [17]). The curve taken locally from the regions assigned to FeO(111) shows a pronounced maximum at the low energy side, indicating the expected dominant presence of Fe^2+^ iron in these regions. The spectrum recorded from the dark grey islands, on the other hand, has a contribution of both components, with Fe^2+^ signal being more pronounced than the Fe^3+^. The plot obtained for the light grey islands is similar but with higher contribution of Fe^3+^ ions. Higher amount of Fe^3+^ in the light grey Fe_3_O_4_ islands indicated that they were thicker than the dark grey islands, so that less (or no) Fe^2+^ signal was detected from the FeO layer underneath the islands (the amount of Fe^2+^ ions per unit volume is higher in FeO than in Fe_3_O_4_). In general, the obtained results are in line with other reports on the growth of iron oxide films on Pt(111) following 1-2 MLs Fe deposition under UHV and post-oxidation in 1 × 10^−6^ mbar O_2_ at 900 K, i.e., they reveal the formation of an FeO(111) layer with a mix-valence Fe_3_O_4_(111) islands of different height nucleating on top of it. The most important observation made was that all the iron oxide structures grown in that way have the same in-plane orientation with respect to the substrate Pt(111).

**Figure 3:**
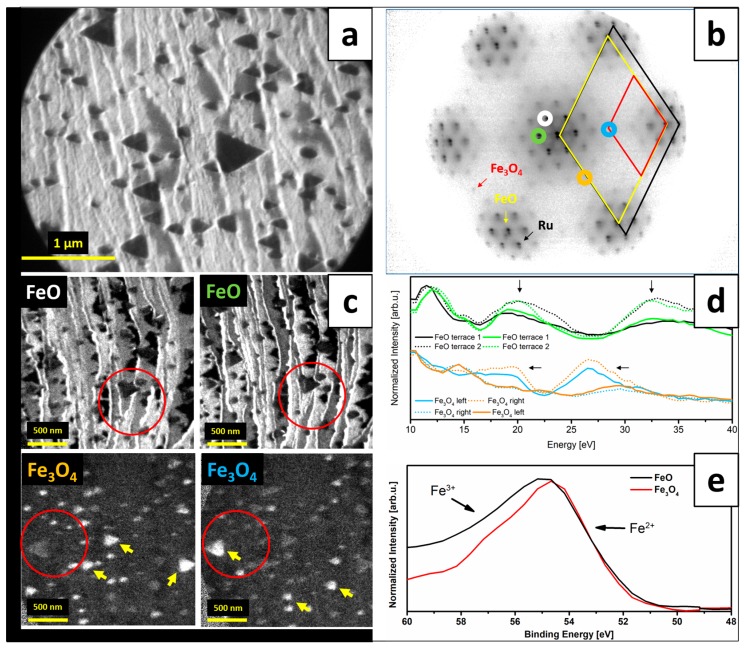
LEEM image (energy: 10 eV) (**a**) and µLEED pattern (energy: 42 eV) (**b**) of iron oxide structures grown on Ru(0001) by ~1.9 MLs Fe deposition under UHV at RT and post-oxidation in 1 × 10^−6^ mbar O_2_ at 900 K; the Ru(0001), FeO(111) and Fe_3_O_4_(111) unit cells are marked on the µLEED pattern with black, yellow and red rhombuses, respectively; (**c**) presents DF-LEEM images (red circles mark the same sample position) obtained by using the diffraction spots of FeO(111) (imaging energy: 20 eV) and Fe_3_O_4_(111) (26 eV; different position than the one at which FeO(111) images were taken) (marked in colors in (**b**)), while (**d**) shows DF-LEEM-IV curves plotted from stacks of DF-LEEM images taken at different energies (the corresponding pairs of curves were normalized and the curves were offset for clarity); (**e**) presents the XPEEM-IV data (photon energy: 150 eV) plotted for various surface regions that correspond to different iron oxide structures.

Deposition of the same amount of iron onto Ru(0001) and post-oxidation resulted in a slightly different sample morphology, as can be seen on the LEEM image in Figure 3a. The µLEED pattern taken from this surface is presented in Figure 3b. Again, LEEM showed bright background with light grey and dark grey islands, while µLEED pattern was a superposition of diffraction spots originating from Ru(0001) ((1 × 1) spots arrangement), FeO(111) ((1 × 1), larger interatomic spacing than that of Ru(0001)), Moiré (satellite spots around the Ru(0001)-(1 × 1) spots) and Fe_3_O_4_(111) ((2 × 2) spots with respect to the Ru(0001)-(1 × 1) spots) (the pattern can be directly compared with the one published in [14], as they were taken at similar beam energy). DF-LEEM images (Figure 3c) revealed that the bright background is FeO(111) and that the dark islands are Fe_3_O_4_(111). The amount of Fe_3_O_4_(111) islands was lower than in the case of Pt(111), however, the average size of the islands was larger. Interestingly, the light grey islands were found not to show any intensity when mapping the FeO diffraction spots and only weak contrast (noise level) at certain energies when mapping the (2 × 2) spots visible in µLEED. In general, the oxidations parameters used should promote, similarly to the case of Pt(111), the growth of bilayer FeO(111) film on Ru(0001) [9] and the appearance of higher order Moiré satellite spots confirmed the presence of such a film in our experiments. However, the amount of deposited iron was not sufficient for the formation of a closed bilayer FeO(111) film fully covering the Ru(0001) substrate. Taking this into account, the light grey islands could be assigned to exposed Ru(0001) and the weak contrast observed when mapping the (2 × 2) LEED spots to oxygen chemisorbed on Ru(0001) (forming the well-known 3O structure [23]).

**Figure 4:**
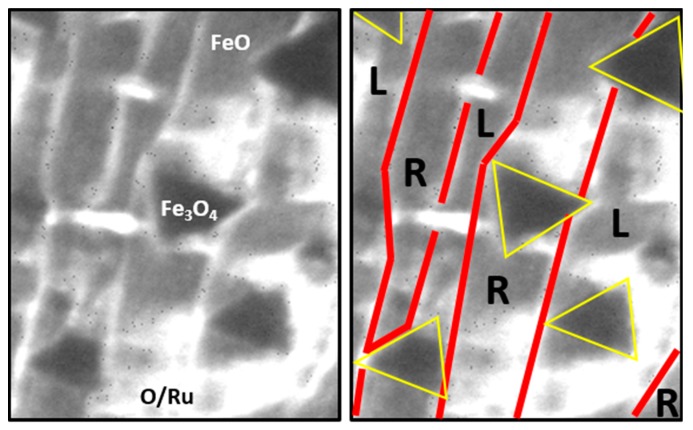
Excerpt from a LEEM image of iron oxides on Ru(0001) showing the 180° rotation of triangular Fe_3_O_4_(111) islands on adjacent monoatomic terraces of the Ru(0001) substrate. The raw image is shown on the left, while the right hand side shows the same image with marked island contours (in yellow) and substrate monoatomic step edges (in red). “L” and “R” indicate terraces with ‘left-oriented’ and ‘right-oriented’ islands, respectively.

The observed Fe_3_O_4_(111) islands had a triangular shape and were similar to those reported in Refs. [6,11,14]. ‘Left-‘ and ‘right-oriented’ triangles could be observed, indicating two possible in-plane orientations of Fe_3_O_4_(111) on Ru(0001). Interestingly, within one substrate terrace, only one islands’ orientation could be seen (Figure 4) (the only exceptions were the biggest islands that were crossing several terraces—in their case, the borders were probably set by step bunches). Dark field images presented in Figure 3c did not only confirm the 180° rotation of Fe_3_O_4_(111) on adjacent Ru(0001) terraces (islands with the same orientation, marked with yellow arrows, show much higher intensity when mapping a particular Fe_3_O_4_ diffraction spot), but also the same rotation of FeO(111) (again, on each substrate terrace only one orientation of FeO(111) was observed). The latter results in the formation of a stripe-like FeO structure on Ru(0001). It has to be mentioned that such a growth mode was already predicted for FeO(111)/Ru(0001) in Ref. [24] based on the six-fold symmetry observed in LEED of few-nanometers-thick FeO(111) films. The 180° rotation is related to the structure of stepped Ru(0001) surfaces, as described in the Introduction (see Figure 1). A single monoatomic Ru(0001) terrace has a three-fold symmetry [10], same as FeO(111) and Fe_3_O_4_(111), therefore it seems intuitive that the epitaxially growing iron oxide will align to the structure of the Ru(0001) substrate (more specifically: to the structure of a particular substrate terrace on which it grows). The rotation of iron oxides is also evident when looking at the DF-LEEM-IV curves taken locally from FeO(111) on adjacent substrate terraces, as well as from left- and right-oriented Fe_3_O_4_(111) islands, using the neighboring FeO(111) and Fe_3_O_4_(111) diffraction spots, respectively (Figure 3d). The DF-LEEM-IV curve obtained for one FeO(111) spot from a particular substrate terrace shows the same character as the curve taken for the other (neighboring) FeO(111) spot from the neighboring terrace. However, the curves taken from the same terrace using different spots are different (the differences are marked with black arrows in Figure 3d). The same holds true for left- and right-oriented Fe_3_O_4_(111) islands and neighboring Fe_3_O_4_(111) diffraction spots. Different character of the curves with respect to those obtained for Fe_3_O_4_(111) on Pt(111) may be due to the fact that the Fe_3_O_4_ islands on Ru(0001) are much thinner and their I–V characteristics may be differently influenced by the underlying substrate. With this respect, it is important to mention that dark field mapping of FeO(111) spots did not show any signal at the location of Fe_3_O_4_ islands, thus confirming the expected transformation of FeO(111) underneath Fe_3_O_4_(111) islands to magnetite [9]. The presence of Fe_3_O_4_(111)/Ru(0001) interface, different from the Fe_3_O_4_(111)/FeO(111) one observed on Pt(111), may have a strong influence on the oxide’s I–V characteristic.

The analysis of the obtained XPEEM data (Figure 3e) revealed that both FeO(111) and Fe_3_O_4_(111) structures consist of a mixture of Fe^2+^ and Fe^3+^ ions. The presence of Fe^+3^ ions in magnetite was expected, however, in wüstite it may only be explained by the presence of iron vacancies in the bilayer FeO film [24].

**Figure 5:**
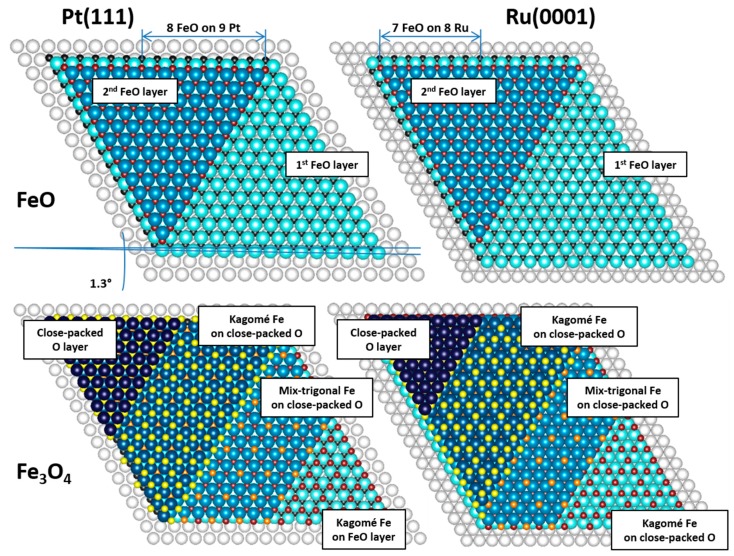
Schematic models of FeO(111) and Fe_3_O_4_(111) structures on Pt(111) and Ru(0001). The atoms in different iron and oxygen sublattices are partially removed to better visualize their different arrangement within different sublayers. The models were made using VESTA 3.4.3 computer software [25].

To better visualize the registries between FeO(111), Fe_3_O_4_(111) and both supports—i.e., Pt(111) and Ru(0001)—we constructed schematic model of the experimentally observed structures and present them in Figure 5. The models take into account the lattice mismatch at different metal-oxide interfaces (FeO(111)/Pt(111), FeO(111)/Ru(0001) and Fe_3_O_4_(111)/Ru(0001)), as well as the rotation angle reported for FeO(111)/Pt(111) [21].

The fact that Fe_3_O_4_(111) islands on both supports exhibit only one in-plane orientation per substrate terrace is believed to be related to (1) the properties of the substrates and (2) the oxidation temperature used. It is well established that the preparation of iron oxide films on Pt(111) and Ru(0001) starts with 1-2 ML Fe deposition and post-oxidation in 1 × 10^−6^ mbar O_2_ at 900–1000 K—the conditions that promote the growth of well-defined FeO(111) wetting layers [8,9]. The oxidation temperature of 900 K favors the formation of bilayer FeO(111) on both supports, which is what we also observe in our experiments. The prolonged oxidation time may, however, result in the transformation of the second FeO(111) layer on Pt(111) to Fe_3_O_4_(111), so that Fe_3_O_4_(111) islands grow on top of the first FeO layer [8], and in the transformation of the bilayer FeO(111) film on Ru(0001) to Fe_3_O_4_(111) (so that Fe_3_O_4_ grows directly on Ru(0001) [11]). Both transformations may potentially occur at the regular terrace sites (and not at the step edges where Fe_3_O_4_ crystallites usually nucleate [8,9]), with Fe_3_O_4_ preserving the same in-plane orientation as the initial FeO. Such growth mode may be driven by various thermodynamic and kinetic factors. Looking at the iron oxides bulk phase diagram [26], the FeO phase should not be thermodynamically stable at the oxidation conditions used in our experiments (1 × 10^−6^ mbar O_2_ and 900 K). However, earlier studies of other authors revealed that in the case of supported iron oxide films the formation of various equilibrium and intermediate surface phases, lying outside of the bulk stability ranges, may be possible [8,9]. On Pt(111), the first FeO layer is stabilized by the strong interaction with the platinum substrate. The second FeO layer can also grow on top of the first layer at certain growth conditions, however, it has a much higher surface free energy and, during prolonged oxidation, undergoes transformation to a more stable Fe_3_O_4_ phase. The growth of Fe_3_O_4_ is, in turn, a transition state towards the final transformation to α-Fe_2_O_3_ (hematite) which, based on the bulk phase diagram, should be the thermodynamically most stable phase at these oxidation conditions. Such transformational order is correlated with an increasing oxidation state of iron (Fe^2+^ in FeO, mixed Fe^2+^/Fe^3+^ in Fe_3_O_4_ and Fe^3+^ in α-Fe_2_O_3_). However, as indicated by the authors of Ref. [26], the complete transformation of Fe_3_O_4_ to α-Fe_2_O_3_ would require oxidation times of several hours (unless a higher oxygen pressure is used). It is thus expected that the observed growth order, i.e., first FeO layer → second FeO layer → transformation of the second FeO layer to Fe_3_O_4_, is related to the relatively low oxidation temperature used, which allows the initial stabilization of the second FeO layer prior to the onset of the Fe_3_O_4_ growth (direct growth of Fe_3_O_4_ islands on the first FeO(111) layer was observed by other authors when the oxidation was carried out at 1000 K [8]), and low oxidation pressure (which makes transformation to α-Fe_2_O_3_ improbable due to the limited oxidation time). In addition, the amount of iron locally available within the second FeO layer is not sufficient for the formation of Fe_3_O_4_ islands that would cover a similar surface area—that is why the nucleating magnetite islands grow at the expense of the neighboring second layer FeO regions which are ‘eaten up’ in the process (the first FeO layer stays intact). The growth of Fe_3_O_4_ may, therefore, lead to the formation of iron vacancies in the FeO regions, which would explain the appearance of the Fe^3+^ component in our XPEEM-IV data. The growth is thus kinetically limited, as it depends on the iron diffusion rate which is lower at lower oxidation temperatures. On Ru(0001), the situation is slightly different, as the initially stabilized form of FeO, i.e., the bilayer Fe-O-Fe-O film, is more weakly interacting with the substrate than the monolayer Fe-O film on Pt(111) and undergoes complete transformation to Fe_3_O_4_ (so that the Fe_3_O_4_ crystallites grow directly on Ru(0001)). The amount of iron locally available for the FeO → Fe_3_O_4_ transformation is twice as large as on Pt(111) and sufficient for the direct transformation. The formation of iron vacancies in the vicinity of the growing islands is also possible, however, not absolutely necessary. The growth is thus not kinetically limited and proceeds more efficiently at the same oxidation conditions.

**Figure 6:**
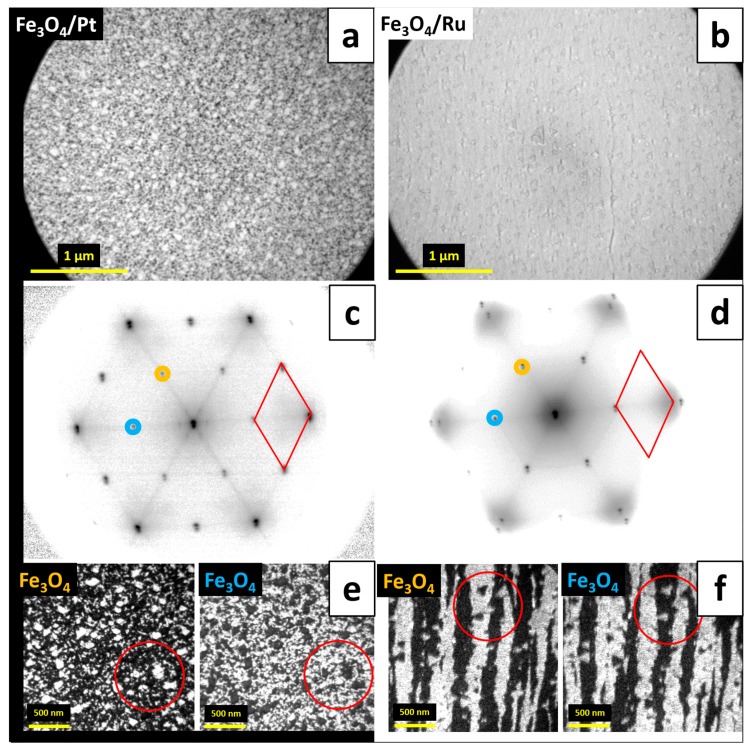
LEEM images (energy: 10 eV) (**a**,**b**) and µLEED patterns (energy: 42 eV) (**c**,**d**) of Fe_3_O_4_(111) films grown on Pt(111) (**a**,**c**) and Ru(0001) (**b**,**d**) by ~5.7 ML Fe deposition (total dose) under UHV at RT and post-oxidation in 1 × 10^−6^ mbar O_2_ at 900 K; the Fe_3_O_4_(111) unit cell is marked on µLEED patterns with red rhombus; (**e**,**f**) present DF-LEEM images obtained by mapping the diffraction spots marked with colors in (**c**) and (**d**), respectively (red circles mark the same sample position on respective images) (imaging energies: 15 (**e**) and 20 eV (**f**)).

In order to verify the influence of the substrate and the growth conditions on the structure of thicker iron oxide films on Pt(111) and Ru(0001), we deposited additional ~3.8 MLs of iron (which resulted in a total iron dose of ~5.7 MLs) onto both supports and oxidized in 1 × 10^−6^ mbar O_2_ at 900 K. Such a procedure typically leads to the proceeding growth of Fe_3_O_4_ [8,9]. The LEEM images obtained for the as prepared samples are shown in Figure 6a,b for iron oxides on Pt(111) and Ru(0001), respectively. The deposited amount of iron was not sufficient for the formation of a closed Fe_3_O_4_(111) film on Pt(111), as magnetite islands tend to coalesce at a total film thickness of about 100 Å on this particular support [8] (where the height of one Fe_3_O_4_(111) unit cell is 4.85 Å). Due to this, we expected the surface to consist of a large number of nucleated Fe_3_O_4_(111) islands with some exposed FeO(111) in between. This morphology can be indeed observed on the LEEM image presented in Figure 6a, which reveals rough surface. The corresponding µLEED pattern, shown in Figure 6c, consists of strong Fe_3_O_4_ reflexes and weak FeO spots—thus supporting the LEEM data. The additional streaks in between the (2 × 2) spots are indicative of small Fe_3_O_4_ domain sizes. On Ru(0001), the deposition–oxidation cycle was expected to promote further transformation of FeO to Fe_3_O_4_. In fact, the LEEM image shown in Figure 6b exhibits uniform contrast, thus indicating that the surface is fully covered with a structurally-uniform iron oxide layer. The µLEED pattern taken from this surface, presented in Figure 6d, revealed the presence of strong Fe_3_O_4_ (2 × 2) reflexes and no diffraction spots originating from FeO. This confirmed that the initial mixed FeO/Fe_3_O_4_ surface transformed, with the help of the additionally deposited iron, into a uniform Fe_3_O_4_ layer. This behavior, different from the one observed on Pt(111), is related to the fact that Fe_3_O_4_ forms much thinner structures on Ru(0001) than on Pt(111) [6] and, therefore, the coalescence of islands may occur for much lower iron dose. To shed more light on the structure of as prepared films, we also performed dark field imaging using the Fe_3_O_4_(111) spots marked on the diffraction patterns shown in Figure 6c,d, which resulted in the DF-LEEM images shown in Figure 6e,f, respectively. Interestingly, two domains rotated by 180° around the [111] axis were observed on both Pt(111) and Ru(0001), resulting in the formation of a patch-like Fe_3_O_4_ layer on Pt(111) and stripe-like magnetite structure on Ru(0001).

**Figure 7:**
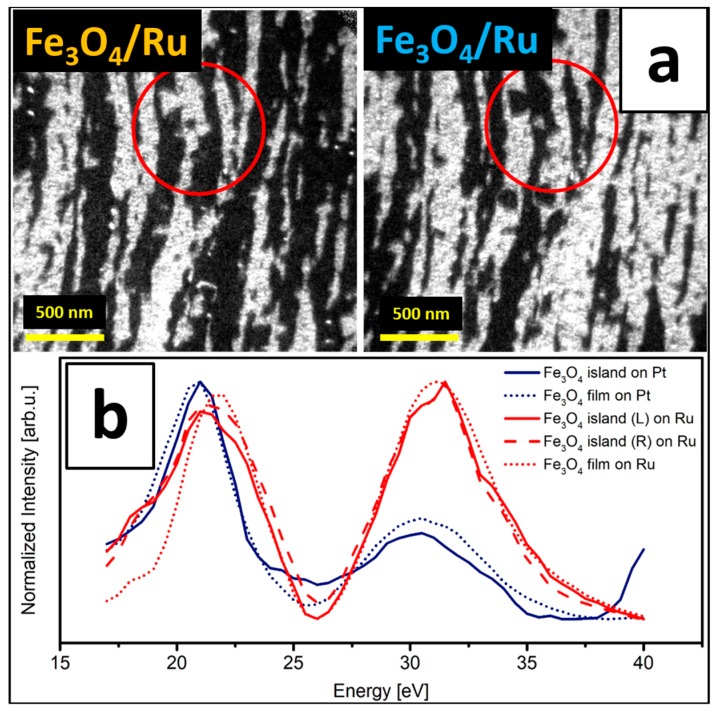
DF-LEEM images of the Fe_3_O_4_(111) film grown on Ru(0001) by ~7.6 MLs Fe deposition (total dose) under UHV at RT and oxidation in 1 × 10^−6^ mbar O_2_ at 900 K (**a**) (red circles mark the same sample position; imaging energy: 26 eV), obtained by mapping the diffraction spots analogous to those marked in colors in Figure 6d; (**b**) shows LEEM-IV curves obtained for Fe_3_O_4_ islands and multilayer films on Pt(111) and Ru(0001).

The appearance of two island orientations on Pt(111) is believed to be related to the presence of newly-nucleated Fe_3_O_4_ islands that did not form via the transformation of the second FeO layer to Fe_3_O_4_, but through the oxidation of the additionally deposited iron. Such islands should have higher probability to grow in one of the two possible orientations, even though one orientation seems to be highly dominant independently on the growth conditions (see e.g. Refs. [8,15]). On Ru(0001), the proceeding transformation of a bilayer FeO(111) film to Fe_3_O_4_(111) results in an iron oxide film with one in-plane orientation per substrate terrace. Only rarely small crystallites with an orientation opposite to that of the terrace on which they grow could be observed (examples of such islands can be seen inside the red circles in Figure 6f). Notably, the stripe-like Fe_3_O_4_(111) structure on Ru(0001) was also observed after the deposition of additional ~1.9 MLs of iron (total iron dose of ~7.6 MLs). The DF-LEEM images obtained for these films by mapping two neighboring Fe_3_O_4_(111) diffraction spots are presented in Figure 7a. This indicates that on this particular support and at these oxidation conditions the oxide grows in a Frank–van der Merwe (layer-by-layer) mode, with subsequent layers adopting the structure of the preceding ones. Such a growth mode favors the stripe-like Fe_3_O_4_ structure also at higher film thicknesses.

Interestingly, the LEEM-IV characteristics obtained for the initial Fe_3_O_4_(111) islands and for the multilayer magnetite films on Pt(111) and Ru(0001) were found to show a very similar character within the energy range of 17–40 eV (Figure 7b). The two peaks, centered at around 21 and 31 eV, were observed for all magnetite structures—regardless of the support and magnetite thickness. In particular, the 31 eV peak was explicitly observed for this iron oxide phase and may be used as a LEEM-IV-fingerprint of Fe_3_O_4_(111). LEEM-IV was already shown to be a reliable method for fingerprinting different iron oxide phases on Pt(111) and Ru(0001), however, usually the energy region of 0–30 eV was used in this respect [11,14,16]. In this region Fe_3_O_4_(111) shows very similar character to FeO(111) (with only a small difference in peak positions), that is why the peak at ~31 eV, which is observed only for magnetite, seems to be a more proper choice for fingerprinting this particular iron oxide phase.

## 4. Conclusions

The results revealed the direct influence of the mutual orientation of adjacent monoatomic terraces of Pt(111) and Ru(0001) on the orientation of FeO(111) and Fe_3_O_4_(111) crystallites epitaxially grown on top of them. The different orientation of terraces originates from different crystal structure (fcc vs. hcp). On cubic Pt(111), which has the same mutual orientation of adjacent monoatomic terraces, FeO(111) grows with the same in-plane orientation on all substrate terraces. For Fe_3_O_4_(111), one or two orientations can be observed depending on the growth conditions. On Ru(0001), the adjacent terraces of which are ‘rotated’ by 180° with respect to each other, the in-plane orientation of the initial FeO(111) and Fe_3_O_4_(111) crystallites is determined by the orientation of the substrate terrace on which they nucleated. Such a growth mode can be explained by the adaptation of three-fold symmetric iron oxides to three-fold symmetric substrate terraces and leads to natural structuring of iron oxide films, i.e., the formation of patch-like magnetite layers on Pt(111) and stripe-like FeO and Fe_3_O_4_ structures on Ru(0001). Even though the orientation of iron oxide crystallites may also depend on other factors, such as the oxidation temperature or substrate-specific growth mode, the observed symmetry-induced structuring provides a general route for tuning the structure and properties of ultrathin [111]-oriented epitaxial films by switching the substrate from fcc(111) to hcp(0001).
